# SY18ΔL60L: a new recombinant live attenuated African swine fever virus with protection against homologous challenge

**DOI:** 10.3389/fmicb.2023.1225469

**Published:** 2023-08-09

**Authors:** Jinjin Yang, Rongnian Zhu, Yanyan Zhang, Jiaqi Fan, Xintao Zhou, Huixian Yue, Qixuan Li, Faming Miao, Teng Chen, Lijuan Mi, Fei Zhang, Shoufeng Zhang, Aidong Qian, Rongliang Hu

**Affiliations:** ^1^College of Veterinary Medicine, Jilin Agricultural University, Changchun, China; ^2^Changchun Veterinary Research Institute, Chinese Academy of Agricultural Sciences, Changchun, China; ^3^Key Laboratory of Prevention & Control for African Swine Fever and Other Major Pig Diseases, Ministry of Agriculture and Rural Affairs, Changchun, China; ^4^Life Science College, Ningxia University, Yinchuan, China

**Keywords:** African swine fever virus, L60L, deleted, attenuated virus, recombinant virus

## Abstract

**Introduction:**

African swine fever (ASF) is an acute and highly contagious disease and its pathogen, the African swine fever virus (ASFV), threatens the global pig industry. At present, management of ASF epidemic mainly relies on biological prevention and control methods. Moreover, due to the large genome of ASFV, only half of its genes have been characterized in terms of function.

**Methods:**

Here, we evaluated a previously uncharacterized viral gene, L60L. To assess the function of this gene, we constructed a deletion strain (SY18ΔL60L) by knocking out the L60L gene of the SY18 strain. To evaluate the growth characteristics and safety of the SY18ΔL60L, experiments were conducted on primary macrophages and pigs, respectively.

**Results:**

The results revealed that the growth trend of the recombinant strain was slower than that of the parent strain in vitro. Additionally, 3/5 (60%) pigs intramuscularly immunized with a 105 50% tissue culture infectious dose (TCID50) of SY18ΔL60L survived the 21-day observation period. The surviving pigs were able to protect against the homologous lethal strain SY18 and survive. Importantly, there were no obvious clinical symptoms or viremia.

**Discussion:**

These results suggest that L60L could serve as a virulence- and replication-related gene. Moreover, the SY18ΔL60L strain represents a new recombinant live-attenuated ASFV that can be employed in the development of additional candidate vaccine strains and in the elucidation of the mechanisms associated with ASF infection.

## Introduction

African swine fever (ASF) is a viral disease associated with high mortality in pigs and has caused considerable harm to the global pig-farming industry. The disease was first identified in East Africa and, subsequently, in other nations of Africa at the beginning of the 20th century. ASF greatly affects the pig industry in epidemic countries (Salguero, 2020). In particular, the 2018 ASF outbreak in China seriously damaged the country’s pig-farming industry (Zhou et al., 2018).

ASFV is a double-stranded DNA virus with an approximate 170–193 kb genome, encoding more than 160 proteins (Wang et al., 2021). ASFV has a complex structure with more than 50 proteins being confirmed as structural proteins (Dixon et al., 2013; Salas and Andrés, 2013; Sánchez-Vizcaíno et al., 2015; Alejo et al., 2018; Dixon et al., 2019). These proteins play an important role in the viral life cycle and are encoded by genes related to viral replication and virulence (Zsak et al., 1998; Tulman and Rock, 2001; Li et al., 2021, 2022; Vuono et al., 2022). The primary functions of other proteins include immune escape and participation in virus pathogenesis. However, the icosahedral structure, including a complex five-layer membrane structure, as well as the large genome of ASFV (Alejo et al., 2018; Liu et al., 2019; Wang et al., 2019) have impeded the in-depth functional characterization of its genes and proteins. As such, no vaccines or therapeutic drugs are currently available for ASF. Nevertheless, numerous studies have been conducted with live gene-deleted vector vaccine candidates. Of particular interest is the identification of virulence-related genes, which are then verified using genetic engineering. For example, the virulence of an ASFV I226R deletion strain was abrogated compared to the parent strain, resulting in the survival of all infected pigs. Hence, the I226R gene was characterized as a virulence gene, the deletion of which creates strains that may prove effective as live vaccine candidates (Zhang Y. et al., 2021). In a similar manner, I177L of the Georgia strain was established as another virulence gene. Moreover, inoculation with the deletion strain ASFV-G-ΔI177L effectively protected all pigs from a viral challenge with the parent strain (Borca et al., 2020). Similarly, the UK, TK, 9GL, and many other genes have all been identified as virulence genes, the deletion of which protects immunized pigs against challenges with the parent strain (Moore et al., 1998; Zsak et al., 1998; Lewis et al., 2000; Zhang Y. et al., 2021). Additionally, the combined deletion of MGF_360-12 L, MGF_360-13 L, MGF_360-14 L, EP153R, and EP402R provides considerable protection against parent strain infection, rendering this a promising vaccine candidate strain (O’Donnell et al., 2015; Xie et al., 2022). Individually, these virulence genes can impact viral morphology, replication, and pathogenicity.

The ASFV L60L gene, located within a variable region, has not been studied in terms of function or toxicity. However, given that relevant deletion strains of the L7L-L11L gene have been generated, the current study focused on L60L of the SY18 strain for further investigation (Zhang J. et al., 2021). The L60L gene of SY18 is located at 4266–4424 nt and encodes 52 amino acids. To identify whether the deletion of the L60L gene affects the replication ability and virulence of the virus, we constructed a deletion mutant (SY18ΔL60L) and evaluated its effect on porcine primary macrophages and on pigs, respectively.

## Materials and methods

### Cells and viruses

Primary porcine alveolar macrophages (PAMs) were obtained from the lungs of pigs without foreign viruses. These 3-month-old pigs were purchased from a local farm with high safety and hygiene standards. After a series of washing processes with phosphate buffer, the cell solutions were centrifuged thrice at 1000 rpm for 10 min per centrifugation. The resulting PAMs were cultured in RPMI (Gibco, Beijing, China) including 10% fetal bovine serum. The incubator conditions for cell culture were set to 37°C and 5% CO_2_. The ASFV SY18 strain (GenBank no. MH766894.2) was separated and preserved by the Changchun Veterinary Research Institute, Chinese Academy of Agricultural Sciences. This experiment used the 8th generation virus that was stored in a biosecurity level 3 (BSL3) laboratory. The median tissue culture infectious dose (TCID_50_) was used to evaluate the virus titer. The virus titer was determined by seeding PAMs in 96-well plates (Corning, Wujiang, China). After the cells formed a monolayer, they were infected with 10-fold serially diluted virus for 5 days. Subsequently, the cells were stained with a p30 monoclonal antibody developed in our laboratory and observed under a fluorescence microscope. Infection with SY18ΔL60L was detected directly by observing red fluorescent cells under a fluorescence microscope. The Reed and Muench methods were used for quantitation (Reed and Muench, 1938).

### Construction and rescue of ASFV recombinant virus SY18ΔL60L

The deletion strain was constructed by homologous recombination method between the parent strain (SY18) genome and the recombinant transfer vector, which contained genes upstream and downstream of the deletion gene L60L (~1200 bp each for the left and right homologous arms), and the L60L gene was replaced by a red fluorescent gene expression cassette with the p72 promoter. The recombinant transfer vector was cloned on the pMD-18T vector by amplifying and connecting the left and right homologous arms. Flanking DNA fragments mapped to the left (1197 bp) and right (1089 bp) of L60L were amplified by polymerase chain reaction (PCR) using SY18 genomic DNA as the template and the following primers: left flank (forward) 5′-AGCAAGAGCCTGCAGAGAA-3′, (reverse) 5′-AAGTATAGTTTATGACACTGAA-3′; and right flank (forward) 5′-CATCCATATCTATTATGAAG-3′, (reverse) 5′-ATGATAAGGACATTATGCTT-3′.

PAMs were prepared in 6-well plates, and each layer was 2 × 10^6^ cells. The cells were then infected with SY18 at MOI of 1. Two hours later, 2 μg of recombinant plasmid pΔL60L-mCherry and 6 μL jetPEI^®^-macrophage transfection reagent (Polyplus, Illkirch, France) were added to each well. Fluorescence was observed after 16 h of incubation. Then the supernatant was discarded, and the cells were washed with PBS 3 times before adding new culture medium, and the cells were repeatedly frozen and thawed for 3 times. Then the cells were diluted in multiple ratios and infected with new cells in a 96 well plate. The wells with more recombinant virus will be selected in the next generation through PCR or fluorescence observation. The deletion strain SY18ΔL60L was obtained after 12 rounds of limited viral dilution.

The following primers were designed for the L60L gene to assess virus purification: forward 5′-TTGATGATTCAGTATTTTGTG-3′ and reverse 5′-TCCTAAACAGATGACTCCAAC-3′. A 137-bp fragment was amplified if the parental SY18 was present.

### Next-generation sequencing

The full-length genome sequence of the deletion strain was verified using next-generation sequencing. Total DNA of the deletion strain SY18ΔL60L and of SY18 were extracted after harvesting infected PAMs (Axygen, USA). DNA (1 μg) was sequenced on an Illumina NovaSeq 6000 and PE150 (Novogene, Tianjin, China).

### *In vitro* growth characteristics of SY18ΔL60L

The virus titers of the SY18 and SY18ΔL60L viruses were assessed in PAM cultures. In brief, PAMs were seeded in a 24-well plate and infected with the virus at an MOI of 0.01. Cell samples were collected 2, 12, 24, 48, 72, 96, and 120 h post-infection (hpi) and subjected to three freeze–thaw cycles. Subsequently, the virus titers were calculated.

### Animal experiments

The pigs weighed 15–20 kg and were purchased from a local farm, which had high biosafety and hygiene standards. Ten pigs were divided into groups A and B (*n* = 5/group). The pigs in group A (ear labels: 76, 77, 78, 12, and 14) were intramuscularly administered 10^5^ TCID_50_ SY18ΔL60L virus; those in group B (vehicle control; ear labels: 01, 02, 03, 04, and 05) were intramuscularly administered 1 mL of normal saline. According to a previous study in our laboratory, the parental wild strain SY18 was sufficient to induce 100% mortality in pigs at a dosage of 10^3^ TCID_50_ (Zhang J. et al., 2021). Therefore, a control group directly challenged with 10^5^ TCID_50_ was not included in this experiment. Following immunization, temperature, food intake, and mental state were monitored daily for 21 days. Additionally, blood and oral and anal swabs were collected on days 0, 3, 7, 14, and 21.

After 21 days, the surviving animals were challenged with the parent strain SY18 (100 TCID_50_). Temperature, clinical manifestations, and survival were monitored daily after the challenge for 21 days. Blood, oral, and anal swabs were collected after 0, 3, 7, 14, and 21 days. The genome copy numbers were determined for each sample using quantitative (q)PCR.

At the end of the 21-day observation period post-challenge, the animals were euthanized with pentobarbital and their internal organs and tissues were excised. The viral load in the heart, liver, spleen, lungs, kidneys, and lymph nodes was determined via detection of the ASFV p72 gene using real-time qPCR. The primer conformed to the WOAH standard. The operation and reaction conditions were as previously described (Zhang J. et al., 2021). The ASFV content in each sample is calculated from the CT value of real-time qPCR, with the formula *y* = −3.1x + 41.2 (y is CT value, x is lg copies/ml). This method was established by our laboratory and is based on plasmids carrying the p72 gene and standard primers of WOAH (data not shown). Additionally, pathological changes in the tissues were analyzed by fixing the heart, liver, spleen, lung, kidney, intestine, and submaxillary lymph node tissues with 4% paraformaldehyde and performing hematoxylin and eosin (HE) staining.

### Detection of ASFV antibodies

To evaluate the immunogenicity of the recombinant viral strain in pigs, the p54 antibody levels in the sera of immunized and infected pigs were quantified using an indirect enzyme-linked immunosorbent assay (ELISA) method developed in our laboratory. The ELISA was performed according to a previously described protocol (Zhang J. et al., 2021). Subsequently, the optical density (OD) at 450 nm was measured using an iMark^™^ Microplate Reader (Bio-Rad, Hercules, CA, USA). The ratio of the OD_450_ for each sample to the OD_450_ of the positive sample was defined as the S/P value. When the S/P value was >0.25, the test sample was defined as positive for the ASFV antibody.

### Biosafety statement and facility

The animal experiments were implemented in a biosafety level 3 (ABSL-3) laboratory. The experiments were approved by the Animal Welfare and Ethics Committee of the Changchun Veterinary Research Institute, Chinese Academy of Agriculture (Review ID: IACUC of CAS-12-2021-011, approved on December 1, 2021). The infection experiment of ASFV was approved by the Ministry of Agriculture and Rural Affairs.

### Statistical analysis

Statistical significance was determined using unpaired, double tailed, and *t*-tests, and results at a *p*-value < 0.05 were considered significant.

## Results

### Construction and rescue of ASFV recombinant virus SY18ΔL60L

**Figure 1:**
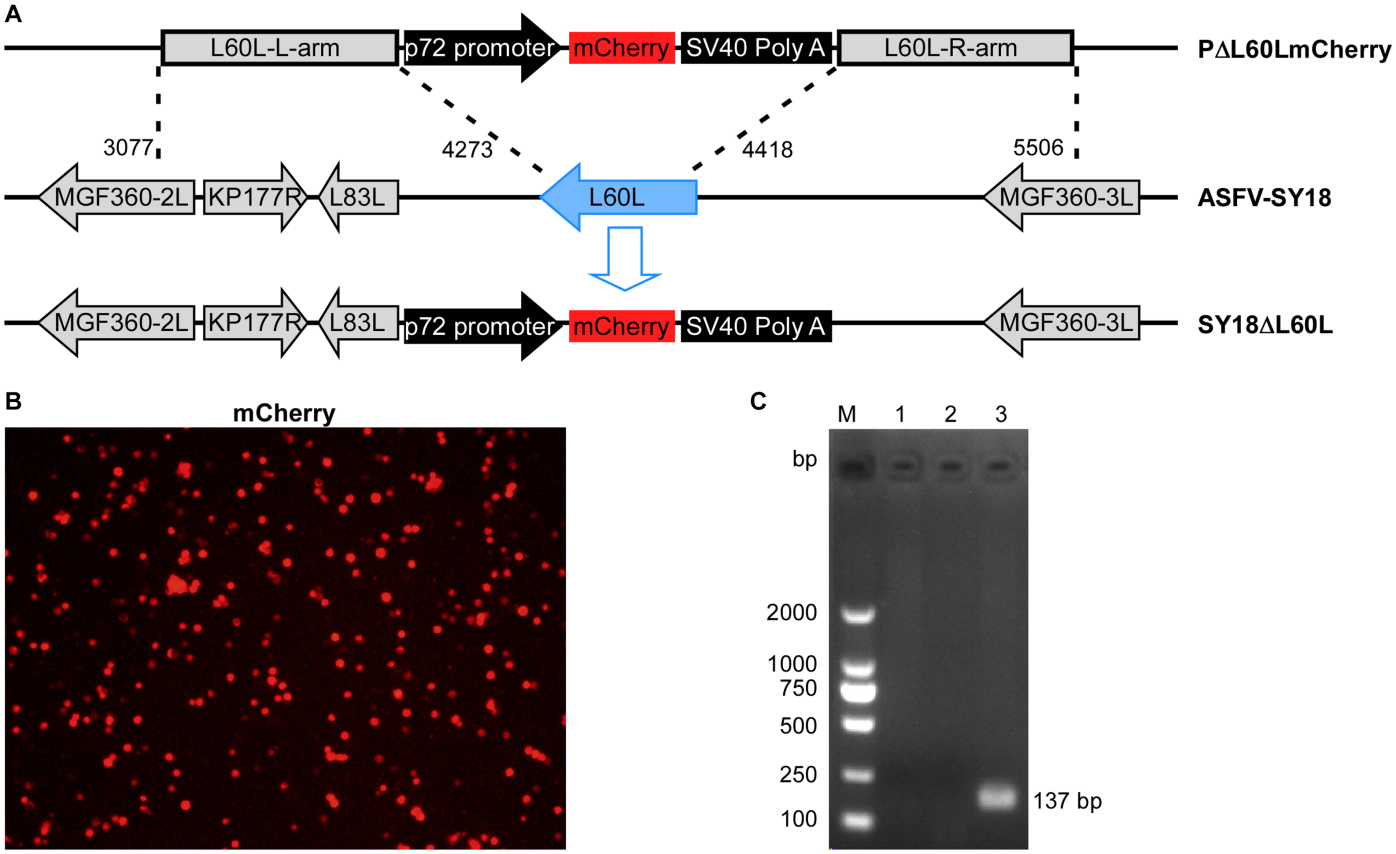
Construction of the L60L deletion recombinant SY18. **(A)** The L60L gene from the SY18 genome was replaced by the p72 mCherryΔL60L recombinant transfer vector via homologous recombination. **(B)** mCherry reporter fluorescence indicates PAM infection with the SY18ΔL60L virus. **(C)** Confirmation of SY18ΔL60L deletion using PCR. Lane 1: L60L gene of SY18ΔL60L, lane 2: control, and lane 3: L60L gene of SY18.

Deletion viruses were generated by homologous recombination between a parental ASFV genome and recombination transfer vectors in PAMs. The recombinant transfer vector contained genes upstream and downstream of the deletion gene L60L, and L60L was replaced by an expression cassette that included a p72 promoter and red fluorescent gene (Figure 1). After the wild-type virus infected the PAMs, the recombinant transfer vector could be transfected to harvest the deletion virus, which emitted red fluorescence. After several rounds of purification, the L60L gene fragment in the genome of the deleted strain was not detected (Figure 1). The accuracy of the deletion strain genome was verified using next-generation sequencing. The results showed that the L60L gene was accurately replaced by the expression box. Compared to the whole-genome sequence of the deletion strain SY18ΔL60L and its parent strain SY18, the genome of the deletion strain did not exhibit significant variation in other unknown areas (GenBank accession number: OR194144).

### Growth characteristics of SY18ΔL60L

**Figure 2:**
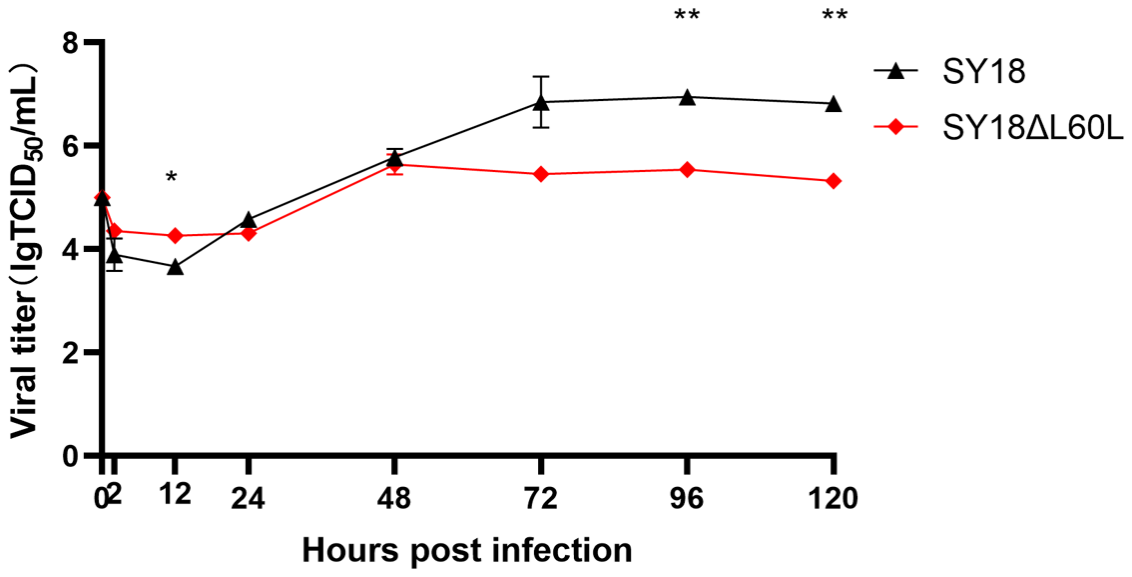
*In vitro* growth characteristics of SY18ΔL60L and SY18. Primary swine macrophage cell cultures were infected (MOI = 0.01) with either of the viruses and the viral yield was titrated at the indicated times post-infection. Data represent the means ± SD from three independent experiments. *p<0.05, **p < 0.01.

The growth curves of SY18ΔL60L and the parental ASFV SY18 were evaluated in PAMs. Virus cultures were collected at different times. The titer of the samples was measured uniformly, and a growth curve was drawn (Figure 2). The results showed that the replication ability of the deletion strain in PAMs significantly decreased (**p < 0.01), with titers that were 10 times lower than that of the parental strain.

### SY18ΔL60L virulence

**Figure 3:**
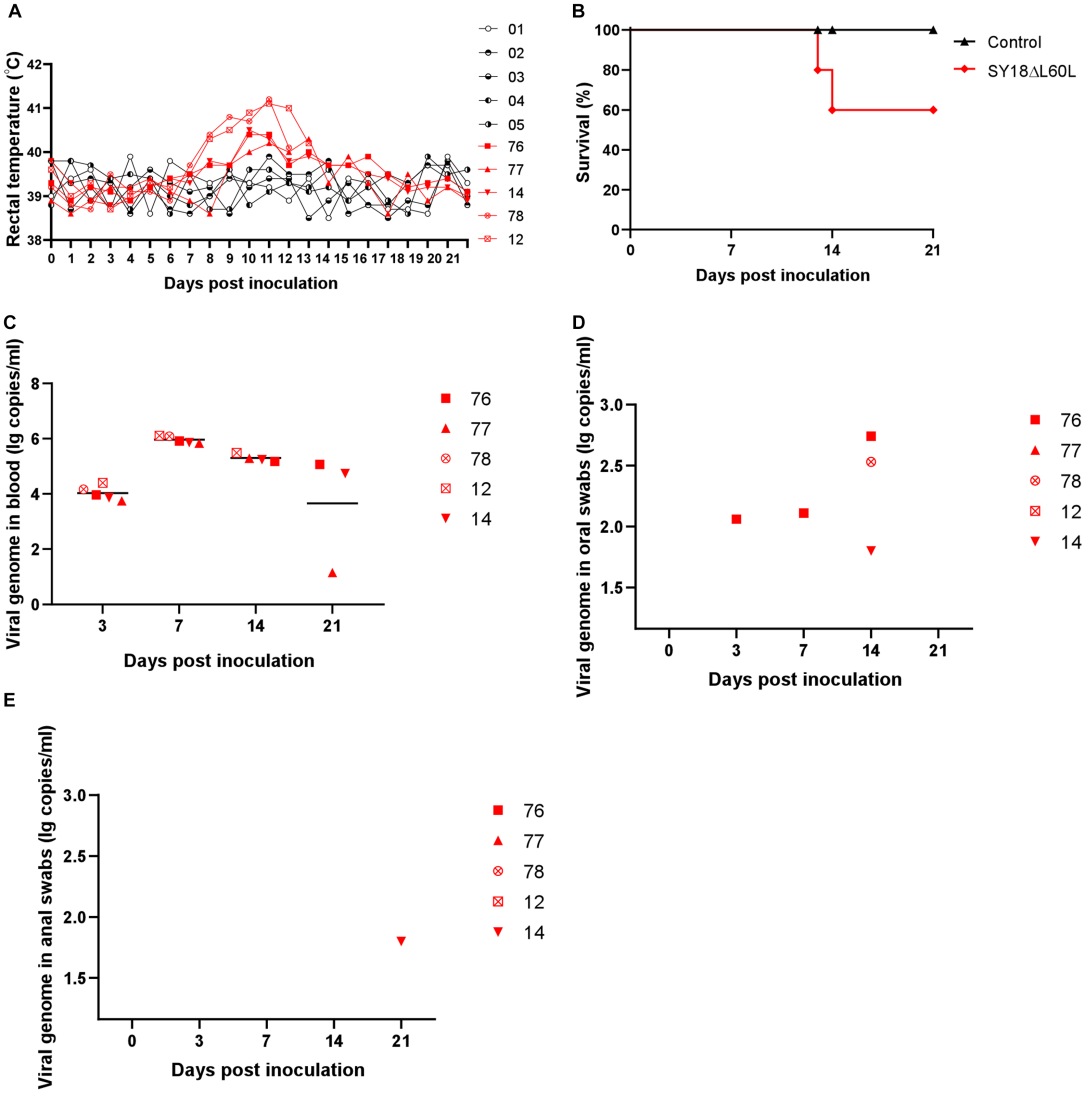
Safety evaluation of SY18ΔL60L immunization. **(A)** Body temperature in the SY18ΔL60L-immunized piglets. **(B)** Percentage of surviving animals. **(C)** Virus titers of blood in immunized piglets. Virus titers of oral **(D)** and anal swabs **(E)** from piglets immunized with the SY18ΔL60L virus.

To verify whether the deletion strain was attenuated, five pigs were intramuscularly immunized with the SY18ΔL60L virus, while the vehicle controls were injected with normal saline. During the 21-day observation period after immunization, two pigs showed clinical features, such as increased body temperature, anorexia, and red patches on their skin until death, whereas three pigs survived. The three surviving pigs showed a small increase in body temperature on day 9 after immunization, but not as much as the pigs that later died. The body temperatures of the three pigs returned to normal on the 14th day after immunization. The virus content in the blood of these three pigs remained at a high level until the 21st day. The virus was detected in oral swabs of 2 pigs on day 14, but the virus was not detected in oral swabs of 3 pigs on day 21 (Figure 3D). The virus was detected in anal swabs from only 1 of 3 survived pigs at day 21, with low content of the virus. The five vehicle control pigs survived without any adverse reactions (Table 1 and Figures 3A,B).

**Table 1:** Swine survival and fever response following immunization with a 10^5^ TCID_50_ dose of SY18ΔL60L.

Group	Before challenge				
	No. of survivors/total	Mean time to death (days ± SD)	Fever		
			No. of days to onset (days ± SD)	Duration No. of days to onset (days ± SD)	Maximum daily temp (°C ± SD)
SY18ΔL60L	3/5	13.5 ± 0.5	11.34 ± 0.47	2.67 ± 1.25	40.93 ± 0.31
Control	5/5	–	–	–	–

Viremia peaked on day 7 post-immunization and subsequently declined until day 21 (Figure 3C). Viral DNA was not detected in the oral swabs of any pigs (Figure 3D). Moreover, the anal swabs for all pigs, save for one, were undetectable for viral DNA. The remaining animal exhibited very low ASFV DNA content on day 21 (Figure 3E).

### Immunoprotective effects of SY18ΔL60L against infection with the parent SY18 strain

**Figure 4:**
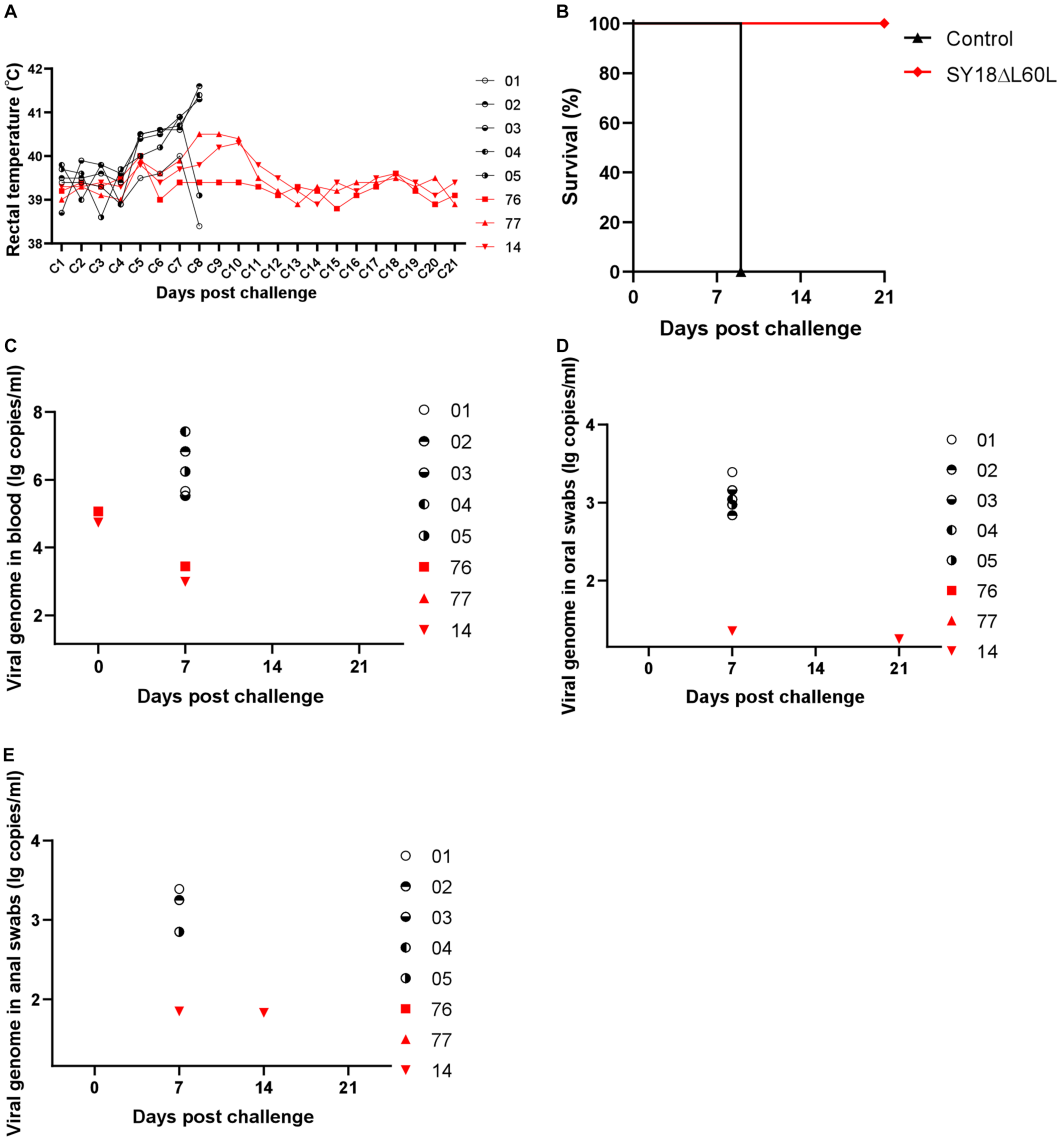
The efficacy of SY18ΔL60L immunization against SY18 infection. **(A)** Rectal temperature in the SY18ΔL60L-immunized piglets challenged with SY18. **(B)** Percentage of animals surviving after challenge. **(C)** Viral DNA levels in piglets of blood immunized with SY18ΔL60L following SY18 virus challenge. Viral DNA in oral **(D,E)** anal swabs from piglets immunized with SY18ΔL60L and challenged with the SY18 virus.

To determine whether the three surviving pigs mounted significant responses to elicit protection against infection by the parent virus strain, a virus challenge experiment was performed with the three pigs that survived immunization and the five vehicle control pigs. Each pig was intramuscularly injected with SY18 (100 TCID_50_). After 21 days of observation, three pigs survived. One of the surviving pigs experienced an instantaneous increase in body temperature but recovered (Table 2 and Figure 4A). All five animals in the control group were euthanized on day 9 post-challenge, while the three pigs immunized with SY18ΔL60L survived until the end of the 21-day observation period (Figure 4B).

**Table 2:** Swine survival and fever response following immunization with SY18ΔL60L and challenge with parental SY18.

Group	Challenge				
	No. of survivors/total	Mean time to death (days ± SD)	Fever		
			No. of days to onset (days ± SD)	Duration No. of days to onset (days ± SD)	Maximum daily temp (°C ± SD)
SY18ΔL60L	3/3	–	8 ± 0	2 ± 0^*^	40.3 ± 0.21
Control	0/5	9 ± 0	5.75 ± 0.83	3 ± 1.22^*^	41.0 ± 0.52

* *p* < 0.05.

Viremia was detected in two of the immunized pigs on days 0 and 7 post-challenge, respectively; however, ASFV DNA was not detectable in any blood samples after day 7 (Figure 4C). Moreover, relatively high levels of ASFV DNA were detected in the oral swabs of the control pigs, whereas oral samples from one immunized pig contained low ASFV DNA levels (Figure 4D). Similarly, the anal samples from one immunized pig tested positive for low levels of ASFV DNA (Figure 4E). Hence, immunization of pigs with the SY18ΔL60L virus offered relative protection against subsequent SY18 ASFV infection.

**Figure 5:**
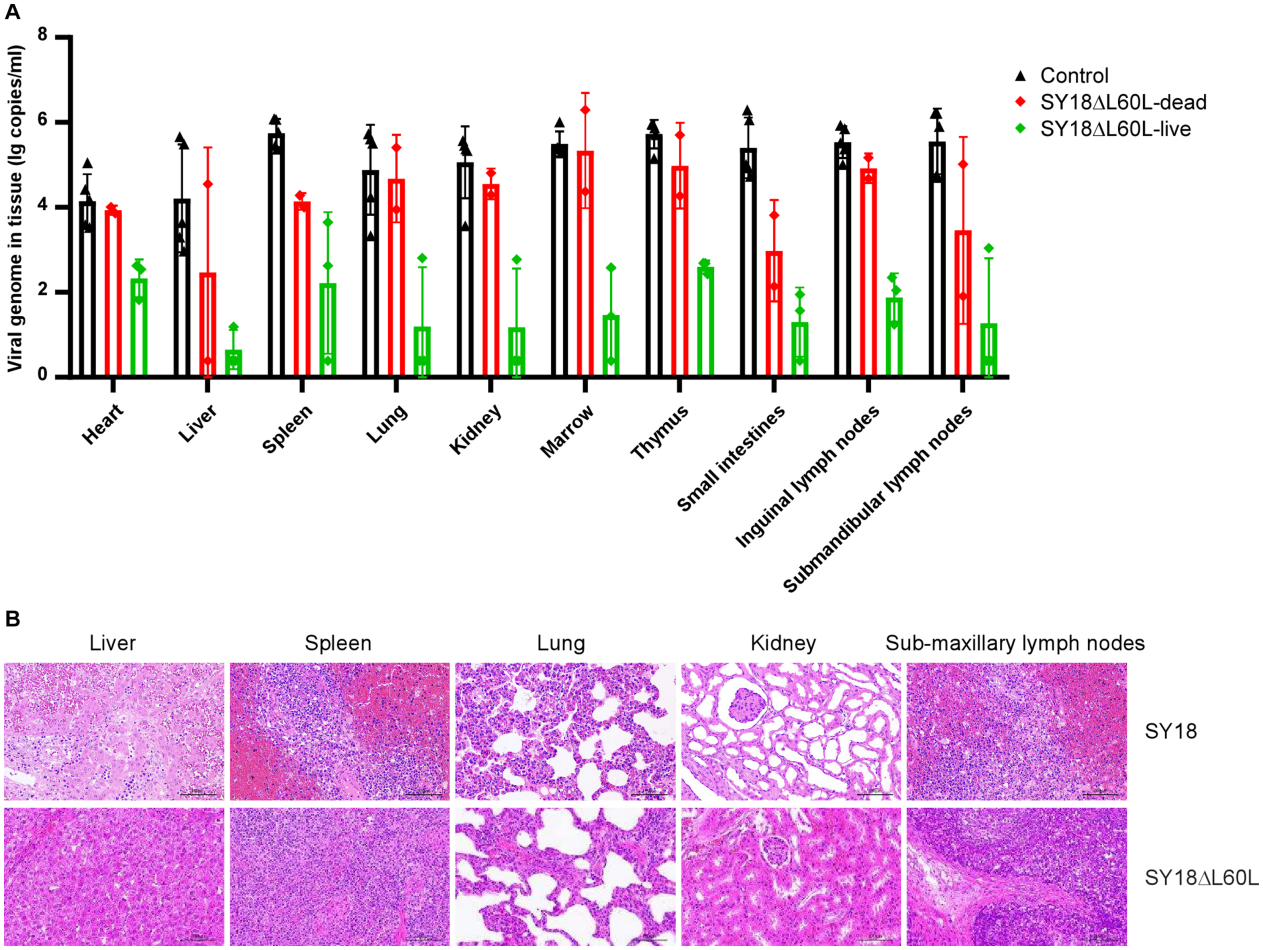
Viral loads and histological changes in various tissues. **(A)** Viral load in the tissues. **(B)** Histopathological analysis of the liver, spleen, lungs, kidneys, and submaxillary lymph nodes in the SY18ΔL60L-immunized and control SY18-infected groups.

Following the 21-day observation period post-virus challenge, the viral load was assessed in the tissues of the control and immunized groups. Results showed that the viral load in all tissues was higher in the virus-challenged control group than in the SY18ΔL60L-immunized group (Figure 5A). Moreover, compared to the immunization group, the liver tissue structure of the control group was slightly abnormal, with expanded hepatic sinuses and increased inflammatory cell infiltration. Additionally, in the control group, the spleens exhibited lesions characterized by severe abnormalities in the tissue structure, atrophy of splenic nodules, and necrosis and degeneration of residual lymphocytes. In contrast, the livers and spleens of the immunized group were normal. Moreover, the lung tissue structure in the control group was slightly abnormal, with alveolar atrophy and collapse and lung parenchyma, whereas that of the immunized group was normal. The renal tissue structure of the control group was also abnormal, with widely expanded renal tubules, atrophic epithelial cells, and an unclear cell border. Meanwhile, the renal tissue structure of the immunized group was relatively normal, without thickening of the basement membrane; however, some renal tubules were slightly expanded. Finally, the structure of the submaxillary lymph nodes was severely abnormal in the control group; the structure of the lymph follicles was not evident, and many lymphocytes were necrotic. In contrast, that of the immunized group was normal without obvious degeneration (Figure 5B).

### Immune response to SY18ΔL60L

**Figure 6:**
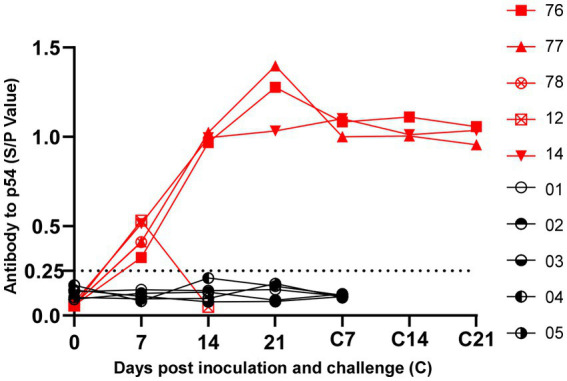
Detection of antibodies against ASFV in pig sera. ASFV specific antibodies levels of Serum were measured using the African Swine Fever Indirect ELISA kit. The 0.25 threshold is represented by the dotted line.

Indirect ELISA was used to detect the levels of anti-ASFV antibodies in pig serum. The pigs in the immune deletion virus group began to show antibody positivity on day 7, and the antibodies in the surviving pigs were at a high level on day 21. After the virus challenge, the antibody levels of the three surviving pigs remained high until 21 days, with an S/P value of approximately 1.0. In contrast, the antibody levels of the non-surviving pigs prior to the virus challenge were lower (Figure 6).

## Discussion

Given the substantial harm to the global pig industry caused by ASFV, research on the genetic function of the virus is continuous. In particular, research on virus virulence genes remains extremely important. Virulence genes, after their deletion, can be used as vaccine candidates to prevent and control ASF epidemics (Zsak et al., 1996; Monteagudo et al., 2017; Borca et al., 2020; Gladue et al., 2021; Zhang J. et al., 2021; Zhang Y. et al., 2021). In addition, the complexity and variability of the ASFV genome have hindered research on the functions of various genes. Studies on the genes that affect the replication, virulence, and interaction mechanisms between the virus and the host may lead to the development of an ASFV vaccine and antiviral drugs and eradication of the ASF epidemic.

Research advances on ASFV have been made regarding the characterization of ASFV virulence genes, leading to the development of candidate live-attenuated vaccines. Nevertheless, the continued investigation and characterization of ASFV genes will not only provide insights regarding the viral replication cycle but also define their roles in viral virulence. Indeed, O’Donnell et al. (2015) generated an avirulent mutant of a highly virulent ASFV strain by deleting six genes, including MGF360 and MGF505. Immunization of pigs with the resulting attenuated strain provided protection against the parental virus strain. Meanwhile, the genomes of certain cell-adapted ASFV strains reportedly lose their MGF360 and MGF505 genes during passage through adaptive cell lines, thus resolving the issue of ASFV-adaptive cell lines (Pires et al., 1997; Krug et al., 2015). That is, establishment of a cell line adapted to a strain will not only solve the problem of vaccine production but also facilitate a more in-depth analysis of ASFV infection and pathogenesis. Encouragingly, a deletion virus based on ASFV-G-ΔI177L has been described that is capable of adapting to cell culture; deletion of the left variable region led to the effective replication of the virus in a stable porcine epithelial cell line (Borca et al., 2021). Hence, deleting other virulence genes on the basis of SY18ΔL60L may generate viral strains capable of effective replication in other cell lines, thus providing support for the production of ASFV vaccines.

In the current study, the L60L gene is a type that has not yet been characterized. The L60L gene of the highly virulent isolate ASFV SY18 was deleted to assess its effect on viral replication in PAMs, as well as determine whether the deletion mutant led to virus attenuation or offered protection against challenge with the parental strain. Hence, PAMs were infected with the deletion mutant SY18ΔL60L or the parental strain SY18. The growth curves showed that the replication ability of the deletion strain was slightly lower than that of the parent strain. This result suggests that the L60L gene may impact SY18 replication *in vitro*. Meanwhile, *in vivo*, the survival rate of pigs 21 days after immunization with the SY18ΔL60L mutant was 60%. However, the surviving pigs exhibited protection against a subsequent challenge with the SY18 parental strain (100 TCID_50_). Importantly, the serum from all three pigs 21 days post-challenge was anti-p54 antibody positive. Further analysis revealed relatively normal pathology of various organs and tissues in the immunized group. Collectively, the *in vivo* results suggest that deletion of the L60L gene can weaken ASFV virulence in pigs. However, the underlying mechanism warrants further investigation. Moreover, the cause of the cytotoxic effects that led to the death of 40% of the study group during the immunization observation period requires elucidation. This experiment had immune mode limitations. By altering immune pathways, such as oral immunization, the survival rate of the immunized group of pigs may increase.

Herein, L60L was characterized as a virulence related ASFV gene. Continued in-depth analysis of this gene might provide novel targets for the development of effective ASFV vaccines and antiviral drugs. The combined deletion of more virulence-related target genes may serve as a viable vaccine candidate for preventing and controlling the ASF epidemic, which will signal a breakthrough for preventing and controlling the ASF epidemic and a substantial advancement of the results of this study.

## Conclusion

In this study, the deletion strain of ASFV L60L gene was constructed by homologous recombination technology. Five pigs were immunized with 10^5^ TCID_50_ dose of recombinant virus, SY18ΔL60L, for virus challenge protection experiment. The results showed that 60% of pigs survive after being immunized against the gene deficient virus, and the surviving pigs are 100% immune to the attack of their parent virus strain. It indicates that the absence of the L60L gene attenuated the virulence of ASFV, which was suggested that the L60L is a virulence-related gene. In addition, it was found that the deletion of L60L gene in ASFV may affect the replication level of virus on PAMs by measuring the growth curves of SY18ΔL60L and SY18 *in vitro*. However, deletion of the L60L gene alone did not enable 100% survival of pigs after immunization, and there were problems with viremia and excretion of the virus during the 21-day observation period, indicating the limitations of this deletion as a vaccine candidate. Fortunately, many studies have shown that the simultaneous deletion of multiple virulence genes increases the safety and potency of the gene deletion virus. Therefore, the deletion of L60L gene combined with deletion of CD2V, MGFs, I226R and other genes has certain potential to solve the safety problem of current vaccine candidate strains. Moreover, it will be helpful to study the related mechanism of L60L affecting virus virulence and replication ability by combining transcriptomic or proteomic techniques to compare the different expression differences of SY18ΔL60L and SY18 infected pig or PAMs.

## Data availability statement

The data presented in the study are deposited in the NCBI repository, accession number OR194144.

## Ethics statement

The animal study was reviewed and approved by the Animal Welfare and Ethics Committee of the Changchun Veterinary Research Institute, Chinese Academy of Agriculture (Review ID: IACUC of CAS-12-2021-011, approved on December 1, 2021) Ministry of Agriculture and Rural Affairs of China.

## Author contributions

RH and AQ designed the experiments. JY, RZ, YZ, JF, XZ, HY, QL, TC, LM, FZ, and SZ performed the experiments. JY, RZ, and YZ analyzed the data. JY and RH wrote the manuscript. All authors contributed to the article and approved the submitted version.

## Funding

This study was funded by the National Key Research and Development Program of China (grant numbers 2021YFD1801400 and 2021YFD1801204).

## Conflict of interest

The authors declare that the research was conducted in the absence of any commercial or financial relationships that could be construed as a potential conflict of interest.

## Publisher’s note

All claims expressed in this article are solely those of the authors and do not necessarily represent those of their affiliated organizations, or those of the publisher, the editors and the reviewers. Any product that may be evaluated in this article, or claim that may be made by its manufacturer, is not guaranteed or endorsed by the publisher.

## Abbreviations

ASF: African swine fever
ASFV: African swine fever virus
HE: hematoxylin and eosin
Hpi: hours post-infection
OD: optical density
PAMs: primary porcine alveolar macrophages
PCR: polymerase chain reaction
ELISA: enzyme-linked immunosorbent assay
qPCR: quantitative PCR
TCID_50_: tissue culture infectious dose
